# Oral Sulforaphane Intervention Protects Against Diabetic Cardiomyopathy in db/db Mice: Focus on Cardiac Lipotoxicity and Substrate Metabolism

**DOI:** 10.3390/antiox14050603

**Published:** 2025-05-16

**Authors:** Pan Wang, Ziling Wang, Xinyuan Jin, Mengdi Zhang, Mengfan Shen, Dan Li

**Affiliations:** 1 Department of Nutrition, School of Public Health, Sun Yat-Sen University, Guangzhou 510080, China; wangp328@mail2.sysu.edu.cn (P.W.); wangzling@mail2.sysu.edu.cn (Z.W.); jinxy8@mail2.sysu.edu.cn (X.J.); zhangmd36@mail2.sysu.edu.cn (M.Z.); shenmf@mail2.sysu.edu.cn (M.S.); 2 Guangdong Provincial Key Laboratory of Food, Nutrition and Health, Guangzhou 510080, China; 3 Guangdong Engineering Technology Center of Nutrition Transformation, Guangzhou 510080, China

**Keywords:** sulforaphane, cruciferae, diabetic cardiomyopathy, diabetes mellitus, lipotoxicity, substrate metabolism, mitochondrial function

## Abstract

The protective effect of cruciferae-derived sulforaphane (SFN) on diabetic cardiomyopathy (DCM) has garnered increasing attention. However, no studies have specifically explored its mechanistic involvement in cardiac substrate metabolism and mitochondrial function. To address this gap, Type 2 diabetes mellitus (T2DM) db/db mice were orally gavaged with vehicle or 10 mg/kg body weight SFN every other day for 16 weeks, with vehicle-treated wild-type mice as controls. SFN intervention (SFN-I) alleviated hyperglycemia, dyslipidemia, HOMA-IR, serum MDA levels, and liver inflammation. Furthermore, SFN-I improved the lipotoxicity-related phenotype of T2DM cardiomyopathy, manifested as attenuation of diastolic dysfunction, cardiac injury, fibrosis, lipid accumulation and peroxidation, ROS generation, and decreased mitochondrial complex I and II activities and ATP content, despite having no effect on ceramide abnormalities. Protein expression data revealed that the model mice exhibited upregulated cardiac CD36, H-FABP, FATP4, CPT1B, PPARα, and PDK4 but downregulated GLUT4, with unchanged MPC1 and MPC2. Notably, SFN-I significantly attenuated the increase in CD36, H-FABP, CPT1B, and PPARα. These results suggest that chronic oral SFN-I protects against DCM by mitigating overall metabolic dysregulation and inhibiting cardiolipotoxicity. The latter might involve controlling cardiac fatty acid metabolism and improving mitochondrial function, rather than promoting glucose metabolism.

## 1. Introduction

Diabetes mellitus (DM) is a devastating metabolic disorder, with a high chance of developing cardiovascular complications, including diabetic cardiomyopathy (DCM). As a primary etiology of heart failure (HF) and associated mortality, DCM has garnered considerable attention since the 1970s, especially during the past decade [1,2]. It is a clinically distinct entity, commonly characterized by impairments in cardiac structure, morphology, and function, independent of macrovascular complications of DM, or valvular, hypertensive, and congenital diseases [1,3]. Cardiac remodeling, fibrosis, hypertrophy, and coronary microvascular defects represent the characteristic phenotypes of DCM. Although not fully understood, multiple systemic and cardiac-local mechanisms contribute to DCM, encompassing hyperglycemia, dyslipidemia, insulin resistance (IR), disturbances in cardiac substrate metabolism, lipotoxicity, glucotoxicity, mitochondrial dysfunction, oxidative stress, inflammation, and endoplasmic reticulum (ER) stress [1,2,3,4]. Currently, conventional therapeutic approaches, mainly focusing on glycemic control, have demonstrated limited efficacy in DCM and related HF [1,2]. Disorders of cardiac glucose and lipid metabolism play key roles in the pathogenesis of DCM. As a highly energy-consuming organ, the heart operates optimally when utilizing a mixture of substrates and possesses certain substrate flexibility to cope with various environmental changes. However, in the context of metabolic cardiomyopathy (MC), including DCM, the myocardium demonstrates a greater preference for fatty acid (FA) utilization (increasing from 60–70% to ~90% of ATP production) at the expense of glucose [1,2,5,6,7,8]. This metabolic remodeling can be adaptive or beneficial in the short term but maladaptive or detrimental in the long term. It may contribute to cardiac structural remodeling and dysfunction by inducing lipotoxicity and mitochondrial dysfunction, decreasing cardiac efficiency, triggering glucotoxicity and redox stress, and interfering with signaling pathways through various metabolites. In particular, the intertwined lipotoxicity and mitochondrial dysfunction constitute the core molecular mechanisms, which originate from and are manifested by lipid accumulation, the formation of lipotoxic byproducts [e.g., ceramide, diacylglycerol (DAG), acylcarnitines, and long-chain acyl-CoAs], and mitochondrial abnormalities (e.g., compromised bioenergetics, ROS generation, uncoupling, fission, Ca^2+^ dyshomeostasis, and apoptosis) [1,2,3,4,7,8,9,10,11,12,13,14]. Therefore, suppression of cardiolipotoxicity through manipulating cardiac substrate metabolism and protecting mitochondrial function represents a promising avenue for DCM management.

Alterations of cardiac metabolism in DCM are likely the result of multiple mechanisms, particularly substrate availability or delivery and deranged expression, translocation, and activities of key molecules in myocardial cell metabolism [7,8,10,11]. The transcellular and/or intracellular transport of FAs is crucial for their utilization, with cluster of differentiation 36 (CD36), heart-type fatty acid-binding protein (H-FABP), and fatty acid transport protein 4 (FATP4) being the major transporters [4,5,6,7]. Correspondingly, glucose uptake is primarily mediated by various glucose transporters (GLUTs), among which GLUT4 is the predominant isoform in the heart that undergoes endocrine and metabolic adjustments [6,7,11]. Mitochondrial FA influx and oxidation rely on the rate-limiting enzyme carnitine palmitoyltransferase 1B (CPT1B), while glucose-metabolized pyruvate is transported into mitochondria via the mitochondrial pyruvate carrier 1/2 (MPC1/2) [15,16]. Both pyruvate dehydrogenase kinase (PDK) and peroxisome proliferator-activated receptor α (PPAR-α) are key hubs for metabolism. PDK determines the metabolic fate of glucose, either for oxidation or glycolysis, by inhibiting pyruvate dehydrogenase (PDH), and may affect glucose and FA oxidation in opposite ways [7]. PPAR-α potentially enhances substrate utilization towards FAs in diseased hearts by distinctly modulating the transcription of multiple genes associated with FA and glucose utilization. Moreover, its cardiac-specific overexpression in mice causes cardiac steatosis and dysfunction, mimicking cardiomyopathy [2,4,7,8,11,14,17,18,19]. Previous studies have shown aberrant cardiac expression of the aforementioned metabolic players in DCM animal models and patients [1,2,5,8,10,11]. Accommodating their expression to alleviate disordered substrate metabolism is proposed to be conducive to improving cardiac function, thereby presenting potential targets for the management of DCM and diabetic HF. Focusing on them to explore or decipher effective lifestyle and pharmacological interventions holds substantial significance. Specifically, phytochemicals, as important components of a healthy diet and a treasure trove for developing natural medicine, have emerged as a promising area of research [20].

Sulforaphane (SFN), an isothiocyanate (ITC)-type phytochemical, is derived from cruciferous vegetables such as broccoli, cauliflower, and cabbage. SFN-rich broccoli sprouts, their extract (BSE), powder, or seeds ameliorate glucose homeostasis, IR, and lipid profiles in patients with type 2 DM (T2DM) or metabolic dysfunction-associated fatty liver disease (MAFLD) (also known as non-alcoholic fatty liver disease, NAFLD) [21,22,23,24], reduce inflammation levels in overweight individuals [25], and improve liver function in male participants with fatty liver [26]. A diet rich in high-glucoraphanin broccoli reduces plasma LDL-C in human subjects [27]. Moreover, preclinical studies have verified that SFN exhibits cardiovascular protective effects, affecting atherosclerosis (AS), myocardial infarction (MI), ischemia-reperfusion injury, and endothelial cell (EC) dysfunction [28]. In addition, animal experiments have confirmed that ITCs including SFN offer protection against DM and its pathogenesis-sharing or similar comorbidities (e.g., obesity and MAFLD), as well as its complications (e.g., DCM, nephropathy, retinopathy, and neuropathy) [28,29,30,31]. When exploring the protective mechanisms of ITCs on cardiometabolic diseases (CMD), liver and adipose tissues are preferred targets due to their pivotal roles in IR and systemic metabolism. In the liver, they positively or negatively regulate gluconeogenesis, lipogenesis, lipid droplet maturation, fatty acid oxidation (FAO), lipophagy, ferroptosis, cholesterol homeostasis and bile acid metabolism, and fibroblast growth factor 21 (FGF21) signaling [32,33,34,35,36,37,38,39,40,41,42]. In adipose tissues, they modulate adipogenesis, white adipose tissue (WAT) browning, and lipolysis [43,44,45,46,47]. Additionally, antioxidation, anti-inflammation, and regulation of gut microbiota are also involved [34,37,38,39,40,41,42].

During the past decade, the impact of ITCs on DCM has garnered increasing attention, with promising findings in several preclinical studies. Initially, Bai and colleagues found that SFN prevented DCM in STZ-induced T1DM FVB mice, which was associated with the activation of nuclear factor erythroid 2-related factor 2 (Nrf2) signaling [48]. Subsequently, the same group further conducted a series of systematic and innovative studies: confirming the preventive effect of SFN in HFD plus STZ-induced T2DM mice and delving deeper into the potential Nrf2-LKB1/AMPK mechanism [49]; extending the Nrf2-mediated efficacy of SFN to SFN-rich BSE in db/db T2DM mice [50]; exploring the partial mediating effect of metallothionein (MT) downstream of Nrf2 in HFD plus STZ-treated mice [51]; discovering better protection from combined SFN and Zn treatment based on Nrf2 and MT in spontaneously developed T1DM OVE26 mice [52]; demonstrating AMPK-mediated lipid metabolism and Nrf2 function in HFD plus STZ-treated AMPKα2-KO mice [53]; identifying ferroptosis as an essential target for preventing DCM by SFN via AMPK/Nrf2 pathways in HFD plus STZ-treated AMPKα2-KO mice and in an ex vivo DCM model [54]. Nevertheless, the relevant field still has unsolved issues, particularly regarding substrate metabolism and mitochondrial functions. Additionally, investigations of oral SFN intervention are scarce. Most importantly, although sharing partial phenotypes and mechanisms, DCM from T2DM and T1DM differs greatly. In T2DM, systemic metabolic disorders and cardiac lipotoxicity play dominant roles in DCM; however, research in this area is extremely limited, and there have been no reports on the effect of SFN on cardiac toxic lipid metabolites.

The objective of our current study is to evaluate the protection conferred by oral SFN administration against DCM in the diabetic db/db mouse model from structural, morphological, and functional perspectives, and to explore mechanistic implications in terms of systemic metabolic improvements (blood biochemistry, adiposity, and MAFLD), as well as local modulation of cardiac substrate metabolism (cardiac abundance of CD36, H-FABP, FATP4, GLUT4, CPT1B, MPC1, MPC2, PDK4, and PPARα) and mitochondrial functions (ATP production and activity of mitochondrial respiratory chain enzymes).

## 2. Materials and Methods

Reagents

R, S-sulforaphane was purchased from LKT Laboratories (S8047, Saint Paul, MN, USA). Oil Red O (ORO) regent and wheat germ agglutinin (WGA) were purchased from Sigma-Aldrich (O1391 and L4895, St. Louis, MO, USA). Dihydroethidium (DHE) and 4′,6-diamidino-2-phenylindole (DAPI) were purchased from Beyotime Biotechnology (S0063 and C1006, Shanghai, China).

Animals and treatments

All animal experimental procedures were approved by the Institutional Animal Care and Ethics Committee (IACUC) of Sun Yat-sen University. Seven-week-old male C57BLKS/J db/db mice and wild-type (WT) control C57BLKS/J mice were purchased from GemPharmatech Co., Ltd. (Nanjing, China) and were housed in the laboratory animal center of Sun Yat-sen University under a constant temperature of 22–26 °C and a 12 h light/dark cycle. All animals had free access to standard diet and water. After two weeks of adaptive feeding, the diabetic db/db mice were included in subsequent experiments and randomly divided into two groups (n = 10–11): the model group (DCM) and the intervention group (DCM + SFN). In addition, age-matched WT male C57BLKS/J mice were used as the control group (Ctrl, n = 11). DCM + SFN mice were orally gavaged with SFN (10 mg/kg) dissolved in 0.5% sodium carboxymethylcellulose (CMC) every other day for 16 weeks. DCM and Ctrl mice were treated with equivalent volumes of the vehicle in the same way. During the experiment, food and water intake and body weight (BW) were daily recorded. After 16 weeks of SFN intervention (SFN-I), blood samples were obtained from the orbital sinus under anesthesia with 1% pentobarbital sodium. Subsequently, the hearts and tibias were isolated and measured, with the ratio of dry heart weight to tibia length (HW/TL) used as one of the parameters of cardiac hypertrophy. Serum samples were collected by centrifugation and stored at −80 °C for further analysis. Cardiac and liver tissues were embedded in optimum cutting temperature (OCT) compound or fixed in 4% paraformaldehyde followed by paraffin embedding. The remaining samples were snap-frozen in liquid nitrogen and stored at −80 °C for indicated analysis.

Measurements of blood pressure and body composition

After 16 weeks of intervention, tail arterial blood pressure, including systolic blood pressure (SBP), diastolic blood pressure (DBP), and mean blood pressure (MBP), was measured in a noninvasive computerized tail-cuff blood pressure meter (Softron Biotechnology, Beijing, China). Body composition was measured using a body composition analyzer (Newmai, Suzhou, China) according to the instrument’s operating instructions.

Echocardiography

To evaluate cardiac function, echocardiography was performed on the mice using the Vevo 3100 Imaging System (Visual Sonics, Toronto, Canada). In brief, mice were placed on a heating plate and anesthetized with isoflurane at concentrations ranging from 1% to 2%, with an oxygen flow rate adjusted between 0.6 and 1.0 L/min. An ultrasound probe was used to find the maximal long-axis section of the mouse heart, then rotated by 90 degrees to locate the maximal short-axis section. The ultrasound image was captured in M-mode (motion mode) when the heart rate of the mice was maintained between 450 and 500 bpm. Next, to obtain the four-chamber incisor, the mice were turned in a head-down, legs-up position, and the probe was directed towards the heart’s apex. The mitral valve blood flow image was captured in PW-mode (pulsed-wave Doppler) at heart rates between 350 and 400 bpm. Vevo LAB 5.6.0 software was utilized to analyze M-mode and PW-mode images. For each mouse, three consecutive cardiac cycles were measured to derive key cardiac indices. They included the mean values of ejection fraction (EF), fractional shortening (FS), early-to-atrial filling ratio (E/A), left ventricular internal diameter during systole (LVIDs) and diastole (LVIDd), and left ventricular posterior and anterior wall thicknesses during systole (LVPWs, LVAWs) and diastole (LVPWd, LVAWd). For PW-Mode images, measurements were taken to determine the mean values of isovolumic contraction time (IVCT), aortic ejection time (AET), isovolumic relaxation time (IVRT), deceleration time (DT), and myocardial performance index (MPI). MPI was calculated as (IVCT + IVRT)/AET.

Serum measurement

Serum levels of fasting blood glucose (FBG), triglycerides (TG), total cholesterol (TC), low-density lipoprotein cholesterol (LDL-C), high-density lipoprotein cholesterol (HDL-C), free fatty acids (FFAs), and malondialdehyde (MDA) were examined using the corresponding commercial reagent kits (Jiancheng, Nanjing, China). Serum fasting insulin (FINS) levels were examined using an enzyme-linked immunosorbent assay (ELISA) (Mercodia, Uppsala, Sweden). Levels of interleukin-6 (IL-6) and tumor necrosis factor-α (TNF-α) were examined using the correspondent ELISA (Meimian, Yancheng, China). All procedures were performed according to the manufacturer’s instructions. HOMA-IR, a common index of IR, was calculated using the following equation: HOMA-IR = [FBG (mmol/L) × FINS (µU/mL)]/22.5.

Histological staining

Paraformaldehyde-fixed heart samples were embedded in paraffin and serially sectioned into 3 µm slices for histological analysis. Then, hematoxylin and eosin (H&E) staining and Sirius red staining were performed routinely following standard procedures. To assess intramyocardial lipid accumulation, ORO staining of cardiac cryosections was carried out. In brief, after being fixed with 4% paraformaldehyde for 15 min, heart-frozen sections (10 µm) were infiltrated in 60% isopropyl alcohol for 10 s, subsequently dyed in ORO solution in the dark at room temperature (RT) for 20 min. Finally, sections were counterstained with hematoxylin solution for 20 s and sealed with glycerin gelatin. Images were captured using a bright-field scanning microscope (KF-FL-400, Ningbo, China) and a light microscope (Olympus BX63, Tokyo, Japan). All images were analyzed using ImageJ 1.54g software.

DHE and WGA staining

To evaluate the superoxide anion production in mouse hearts, frozen sections were stained with 5 µM DHE solution in the dark at 37 °C for 30 min after fixation with 4% paraformaldehyde for 15 min. After washing 3 times with PBS, the sections were counterstained with DAPI solution for 5 min and sealed with an antifluorescent quencher. Myocardial hypertrophy was evaluated using WGA staining of the paraffin sections. In brief, after dewaxing to water and antigen retrieval, the sections were incubated with a 5 µg/mL WGA solution in the dark at 37 °C for 60 min. Both types of stained sections were immediately observed under a fluorescence microscope (Olympus BX63, Tokyo, Japan). Quantitative analysis was carried out using ImageJ 1.54g software.

Myocardial adenosine triphosphate (ATP) content and mitochondrial complex activity

The ATP content in cardiac tissue was measured using the ATP Detection Kit from Beyotime Biotechnology (Shanghai, China) via a fluorescein–luciferase assay. Specifically, 20 mg of heart tissue was thoroughly homogenized with 200 µL of lysis buffer. After homogenization, the mixture was centrifuged at 12,000× *g* for 5 min at 4 °C. The supernatant was collected, with a portion reserved for protein concentration determination. The remaining supernatant was added to the ATP detection working solution according to the kit’s instructions, and the ATP level in the heart tissue was assessed by referring to the ATP standard curve. The results were then normalized to the concentration of tissue proteins to account for any variability in sample protein content.

The activity of mitochondrial respiratory chain complexes I and II in cardiac tissue was assessed via the spectrophotometric method using the Mitochondrial Respiratory Chain Complex I and Complex II Assay Kits, both supplied by Elabscience Biotechnology (Wuhan, China). Initially, heart tissue was homogenized with the corresponding extraction solution to prepare a 10% heart tissue homogenate. Mitochondria were then isolated using a differential centrifugation method. Subsequently, part of the mitochondrial supernatant was used to measure protein concentrations, while the remainder was sequentially treated with the corresponding substrates, inhibitors, or reactants as per the kit’s protocol for further experiments. Finally, the reaction mixture was measured for absorbance at specific wavelengths: 340 nm for complex I and 600 nm for complex II. The final result was normalized to the concentration of mitochondrial proteins.

Detection of cardiac levels of ceramide, DAG, and MDA

Myocardial tissue samples were excised and immediately immersed in ice-cold normal saline solution at a weight-to-volume ratio of 1:9. The tissue was then thoroughly homogenized using a tissue grinder under low-temperature conditions to preserve the integrity of the cellular components. Following homogenization, the samples were centrifuged at 1000× *g* for 20 min. The supernatant was carefully collected and subsequently used for quantification. According to the respective manufacturers’ protocols, levels of ceramide and DAG were examined using ELISA kits (Mlbio, Shanghai, China), and MDA was examined using commercial reagent kits (Jiancheng, Nanjing, China).

NAFLD Activity Score (NAS) and hepatic tissue measurements

Paraffin-embedded liver tissues were cut into approximately 3 µm sections and stained with H&E for general morphological observations. The liver histology was graded using NAS, including steatosis (<5% = 0; 5–33% = 1; 34–66% = 2; >66% = 3), inflammation (none = 0; <2 foci = 1; 2–4 foci = 2; >4 foci = 3), and hepatocellular ballooning (none = 0; few = 1; prominent = 2). Mice with NAS ≥ 5 were correlated with a diagnosis of nonalcoholic steatohepatitis (NASH), and biopsies with scores of less than 3 were diagnosed as “not NASH.” For tissue sample assays, 50 mg of liver tissue was homogenized in 1 mL of lysis buffer using an electric tissue homogenizer. Following a 10 min resting period, the supernatant was collected and used for the determination of liver TG and TC levels according to the manufacturer’s protocol (Applygen, Beijing, China). The levels of IL-6 and TNF-α in the liver were examined using ELISA kits after homogenizing with saline solution (Meimian, Yancheng, China).

Western Blotting

Heart tissues were lysed with RIPA buffer (Beyotime, Shanghai, China) to extract the total protein. Total protein concentrations were determined using the Pierce BCA Protein Assay Kit (Thermo Fisher, San Jose, CA, USA). Equal amounts of protein (15–20 µg) were resolved by 10–15% SDS–PAGE gels and then transferred onto polyvinylidene fluoride (PVDF) membranes (Millipore, Billerica, MA, USA). After blocking with 5% nonfat milk for 2 h, the following primary antibodies were used: Anti-CD36 (1:1000, Abcam, ab200353), Anti-H-FABP (1:2000, Abcam, ab133585), Anti-FATP4 (1:1000, Abcam, ab252923), Anti-CPT1B (1:1000, Abclonal Technology, A6796), Anti-PPARα (1:1000, Santa Cruz Biotechnology, sc-398394), Anti-GLUT4 (1:2000, Santa Cruz Biotechnology, sc-53566), Anti-PDK4 (1:1000, Abclonal Technology, A13337), Anti-MPC1 (1:1000, Cell Signaling Technology, 14462S), Anti-MPC2 (1:1000, Cell Signaling Technology, 46141S), and Anti-β-actin (1:1000, Santa Cruz Biotechnology, sc-47778). The immunoreactive bands were detected using horseradish peroxidase-conjugated secondary antibodies (Santa Cruz Biotechnology, Dallas, TX), and visualized using the ECL detection kit (Thermo Fisher, San Jose, CA, USA). Densitometric analysis was performed for quantification using ImageJ 1.54g software.

Statistical analysis

The data are expressed as the mean ± SEM. Statistical analyses were performed using GraphPad Prism Version 8.0. One-way analysis of variance (ANOVA) followed by Bonferroni’s multiple comparisons test was used. A *p*-value of <0.05 was used to indicate a statistically significant difference.

## 3. Results

### 3.1. SFN-I Improves Systemic Metabolism but Not the Three Common Diabetic Symptoms in db/db Mice

**Figure 1:**
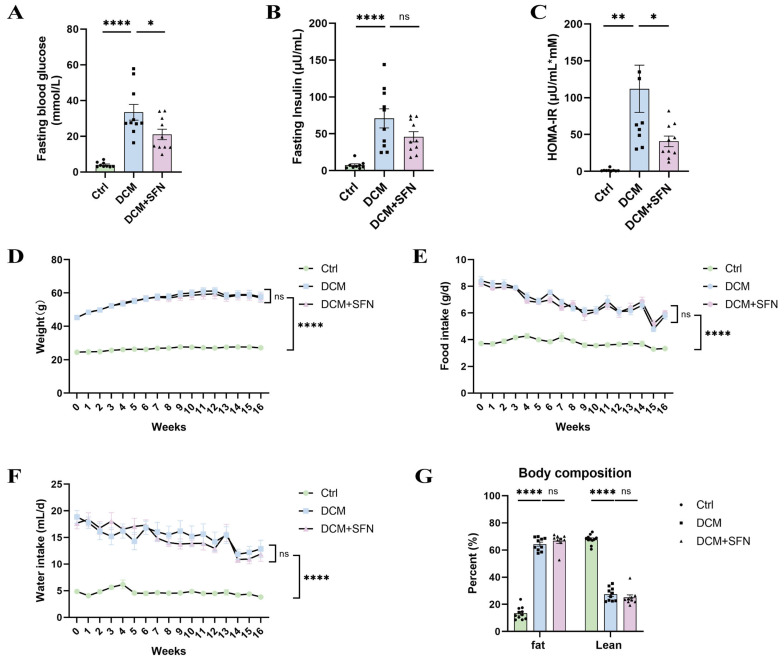
SFN-I improves diabetic metabolic phenotype but not the three symptoms in db/db mice. (**A**–**C**) Serum levels of fasting glucose and insulin, and HOMA-IR at week 16. (**D**) Body weights were monitored weekly throughout the 16-week intervention period. (**E**,**F**) The daily food and water intake of each group of mice was statistically analyzed on a weekly basis for a total of 16 weeks. (**G**) The percentage of body fat and lean mass at week 16. Data are expressed as the mean ± SEM (n = 9–11). * *p* < 0.05, ** *p* < 0.01, **** *p* < 0.0001: significantly different as indicated; ns: not significantly different. HOMA-IR, homeostatic model assessment for insulin resistance; Ctrl, control; DCM, diabetic cardiomyopathy; SFN, sulforaphane.

To obtain a disease model of DCM and evaluate the effect of SFN-I, we used 9-week-old db/db mice and implemented an intervention that lasted for 16 weeks. At the end of the experiment, the model group showed a significant increase in FBG (Figure 1A), fasting insulin levels (Figure 1B), and HOMA-IR (Figure 1C), whereas SFN-I induced a reduction in these parameters, with the differences in FBG and HOMA-IR being statistically significant (Figure 1A–C). In addition, the model mice displayed dyslipidemia characterized by elevated levels of serum FFAs, TC, TG, and LDL-C compared with the control group. However, SFN-I significantly mitigated the increase of FFAs and TC, and induced a decreasing trend in TG and LDL-C. Meanwhile, the HDL-C levels in the model mice were significantly increased after SFN-I (Table 1). The serum MDA concentration in the model group was approximately twice that of the control group, and SFN-I significantly decreased it. Yet, there were no significant differences in serum levels of IL-6 and TNF-α among the three groups (Table 1). During the whole intervention period, db/db mice in the model group exhibited severe and stable diabetic symptoms, including increased BW, polyphagia, and polydipsia, which showed no appreciable improvement after SFN-I (Figure 1D–F). Besides, the diabetic mice had a significantly higher proportion of fat mass and a considerably lower proportion of lean mass, as determined by body composition analysis at the end of the experiment. This profound alteration in body composition under diabetic conditions was unaffected by SFN-I (Figure 1G).

### 3.2. SFN-I Reduces Liver Inflammation Without Significantly Affecting Liver Lipid Deposition in db/db Mice

**Figure 2:**
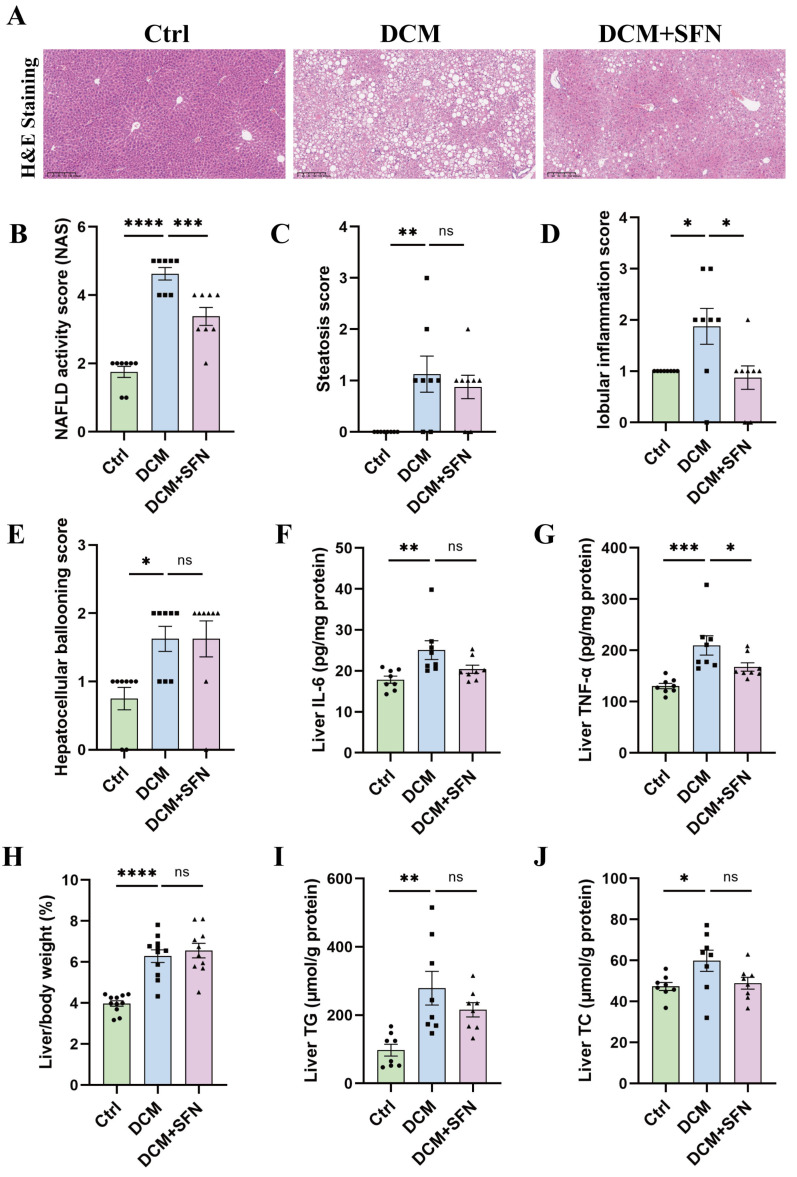
SFN-I reduces liver inflammation without significantly affecting liver lipid deposition in db/db mice. (**A**,**B**) Representative H&E-stained liver images (scale bar = 100 μm) and NAS scores. (**C**–**E**) Individual scores for steatosis, lobular inflammation, and hepatocellular ballooning. (**F**,**G**) Hepatic content of IL-6 and TNF-α. (**H**) The ratio of liver weight (g) to body weight (g). (**I**,**J**) Hepatic content of TG and TC. Data are expressed as the mean ± SEM (n = 8–11). * *p* < 0.05, ** *p* < 0.01, *** *p* < 0.001, **** *p* < 0.0001: significantly different as indicated; ns: not significantly different. TG, total triglycerides; TC, total cholesterol; H&E, hematoxylin and eosin; IL-6, interleukin-6; TNF-α, tumor necrosis factor-alpha; Ctrl, control; DCM, diabetic cardiomyopathy, SFN, sulforaphane.

The db/db model mice had significantly higher NAS scores than the WT mice, with over half achieving a score of 5, a cutoff for NASH. Importantly, SFN-I led to a pronounced reduction in NAS scores (Figure 2A,B). Specifically, the DCM group displayed pronounced steatosis, lobular inflammation, and hepatocellular ballooning compared to the control group. SFN-I effectively alleviated lobular inflammation but had no significant effect on steatosis or hepatocellular ballooning (Figure 2C–E). Quantitative analysis of liver inflammatory factors indicated significantly elevated IL-6 and TNF-α levels in the db/db model mice compared with the control mice. SFN-I effectively reduced TNF-α but had no significant effect on IL-6 (Figure 2F,G). Compared to the control group, the db/db model mice also exhibited a significantly higher liver-to-body weight ratio. This ratio did not change significantly with SFN-I (Figure 2H). Additionally, there were significantly elevated levels of liver TG and TC in the db/db model mice. Although SFN-I tended to reduce them, the differences were not statistically significant (Figure 2I,J).

### 3.3. SFN-I Attenuates Diastolic Dysfunction in db/db Mice

**Figure 3:**
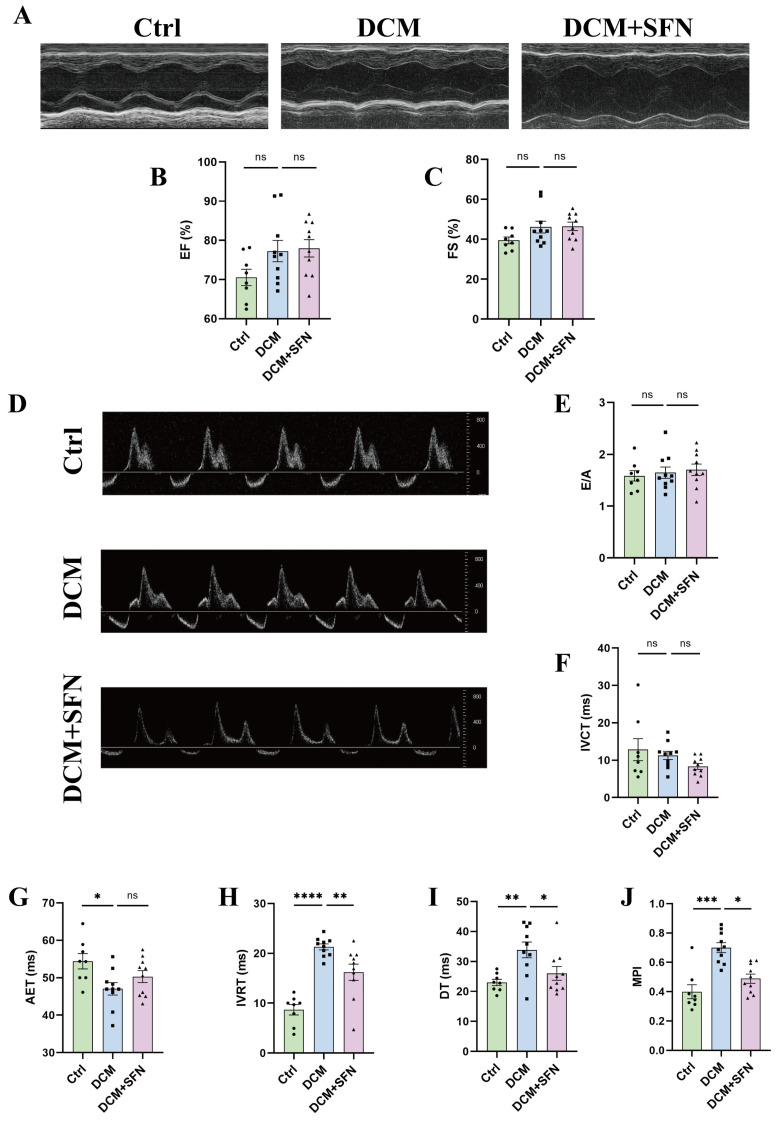
SFN-I attenuates diastolic dysfunction in db/db mice. (**A**,**D**) Representative M-mode and PW-mode images of echocardiography. (**B**,**C**) Measurements of EF (%) and FS (%) based on M-mode. (**E**–**J**) Measurements of E/A, IVCT, AET, IVRT, DT and MPI based on PW-mode. Data are expressed as the mean ± SEM (n = 8–10). * *p* < 0.05, ** *p* < 0.01, *** *p* < 0.001, **** *p* < 0.0001: significantly different as indicated; ns: not significantly different. EF, ejection fraction; FS, fractional shortening; E/A, Early-to-Atrial filling ratio; IVCT, isovolumic contraction time; AET, aortic ejection time; IVRT, isovolumic relaxation time; DT, deceleration time; MPI, myocardial performance index; Ctrl, control; DCM, diabetic cardiomyopathy; SFN, sulforaphane.

The results of echocardiography are presented in Figure 3 and Table 2, with Figure 3A,D showing the original images captured in M-mode and PW-mode, respectively. Compared to the control group, the mice in the model group exhibited a significantly shortened AET, as well as markedly prolonged IVRT and DT, while EF, FS, E/A, and IVCT remained unchanged. The cardiac dimensional parameters, including left ventricular internal diameter (LVIDs and LVIDd) and wall thickness (LVPWs, LVPWd, LVAWs, and LVAWd), did not differ significantly between the control group and the model group. These data suggest that, at the end of the experiment, the diabetic mice exhibited preserved systolic function but impaired diastolic function, with no evident ventricular dilation or hypertrophy. The 16-week SFN-I significantly improved cardiac diastolic dysfunction, as evidenced by a notable reduction in the IVRT and DT values (Figure 3H andFigure 3I, respectively). Besides, MPI, as a comprehensive index that incorporates both systolic and diastolic ventricular function, was higher in db/db mice than in control mice, which was also correspondingly attenuated by SFN-I (Figure 3J). Regarding blood pressure, there were no significant differences in SBP, DBP, and MBP among the three groups. Heart rate was also the same (Table 3).

### 3.4. SFN-I Mitigates Cardiac Injury and Fibrosis in db/db Mice

**Figure 4:**
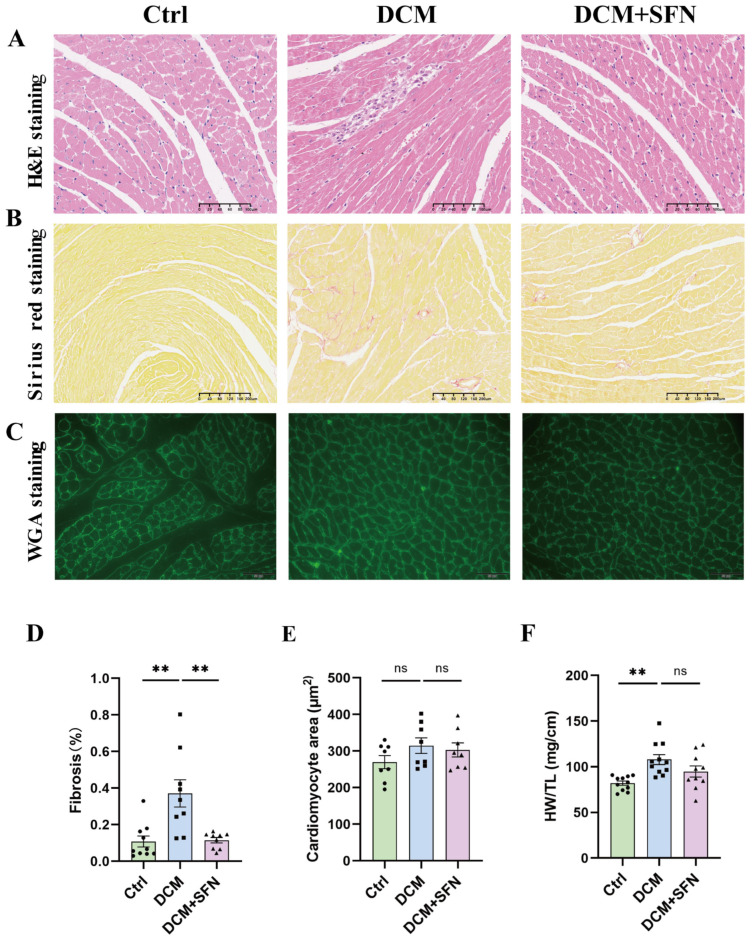
SFN-I mitigates cardiac injury and fibrosis in db/db mice. (**A**–**C**) Representative heart sections with H&E staining (scale bar = 100 μm), Sirius red staining (scale bar = 200 μm), and WGA staining (scale bar = 50 μm). (**D**,**E**) Quantification of myocardial fibrosis and cardiomyocyte cross-sectional area based on Sirius red staining and WGA staining, respectively. (**F**) The ratio of heart weight (mg) to tibial length (cm) (HW/TL). Data are expressed as the mean ± SEM (n = 8–11). ** *p* < 0.01: significantly different as indicated; ns: not significantly different. H&E, hematoxylin and eosin; WAG, wheat germ agglutinin; Ctrl, control; DCM, diabetic cardiomyopathy; SFN, sulforaphane.

To further assess the cardiac structural changes in the db/db mouse model and evaluate the effect of SFN-I, paraffin-embedded heart sections were subjected to histological staining with H&E, Sirius red, and WGA. As shown in Figure 4A, left panel, the myocardial tissue of the control WT mice exhibited a regular and compact cardiomyocyte arrangement with clearly visible nuclei. In contrast, the myocardial tissue of the db/db model mice displayed characteristic pathological features associated with DCM, including disordered arrangement of cardiomyocytes and infiltration of inflammatory cells (Figure 4A, middle panel). Notably, these pathological changes were significantly alleviated by SFN-I (Figure 4A, right panel). Sirius red staining indicated that larger areas of collagen were positively stained in the hearts of diabetic mice compared with those in control mice, which was significantly reduced by 69% following SFN-I (Figure 4B,D). Cardiac hypertrophy was assessed using HW/TL and cardiomyocyte cross-sectional area measured from WGA staining. The quantitative analysis revealed that both indicators increased in the db/db model mice, but only the difference in HW/TL showed statistical significance. Although SFN-I led to a slight reduction in HW/TL, this change did not reach statistical significance. Additionally, no significant difference was established in cardiomyocyte cross-sectional area between db/db mice with and without SFN-I (Figure 4C,E,F).

### 3.5. SFN-I Reduces Cardiac Lipid Accumulation and Oxidative Stress Without Significantly Altering Ceramide and DAG Levels in db/db Mice

**Figure 5:**
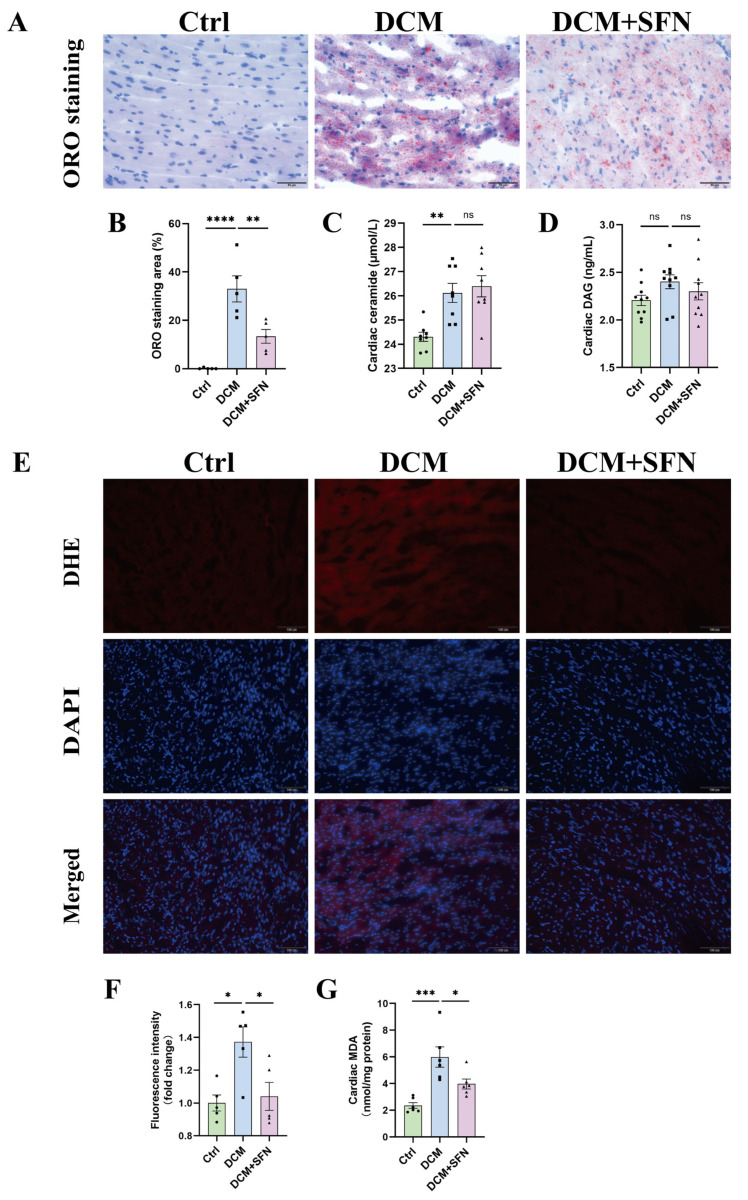
SFN-I reduces cardiac lipid accumulation and oxidative stress without significantly altering ceramide and DAG levels in db/db mice. (**A**,**B**) Representative ORO staining of hearts and quantification of lipid accumulation (scale bar = 50 μm). (**C**,**D**) Cardiac levels of ceramide and DAG. (**E**,**F**) Representative fluorescence images of DHE-stained hearts (scale bar = 100 μm) and quantification of the fluorescence intensity. (**G**) Cardiac levels of MDA. Data are expressed as the mean ± SEM (n = 5–8). * *p* < 0.05, ** *p* < 0.01, *** *p* < 0.001, **** *p* < 0.0001: significantly different as indicated; ns: not significantly different. ORO, Oil Red O staining; DAG, diacylglycerol; DHE, dihydroethidium; MDA, malondialdehyde; Ctrl, control; DCM, diabetic cardiomyopathy; SFN, sulforaphane.

Upon ORO staining, the hearts of WT mice exhibited few visible lipid droplets, while those of the db/db model mice displayed a substantial area of red-stained lipid droplets. This pronounced deposition of lipids was significantly suppressed by SFN-I, with an inhibitory rate of approximately 59% (Figure 5A,B). Subsequently, the cardiac levels of ceramide and DAG, two key lipotoxic mediators, were determined. A higher abundance of both of them was observed in diabetic mice, although this was statistically significant only for ceramide. Neither ceramide nor DAG levels exhibited significant alteration in response to SFN-I (Figure 5C,D). In addition, the production of superoxide anions, as assessed by the fluorescence intensity of DHE staining as well as MDA levels, was significantly elevated in heart tissues of db/db mice relative to those of control mice; SFN-I resulted in a dramatic improvement in both indices (Figure 5E–G).

### 3.6. SFN-I Improves Cardiac Mitochondrial Function and Modulates Key Regulators of FA Metabolism, Rather than Glucose Metabolism, in db/db Mice

**Figure 6:**
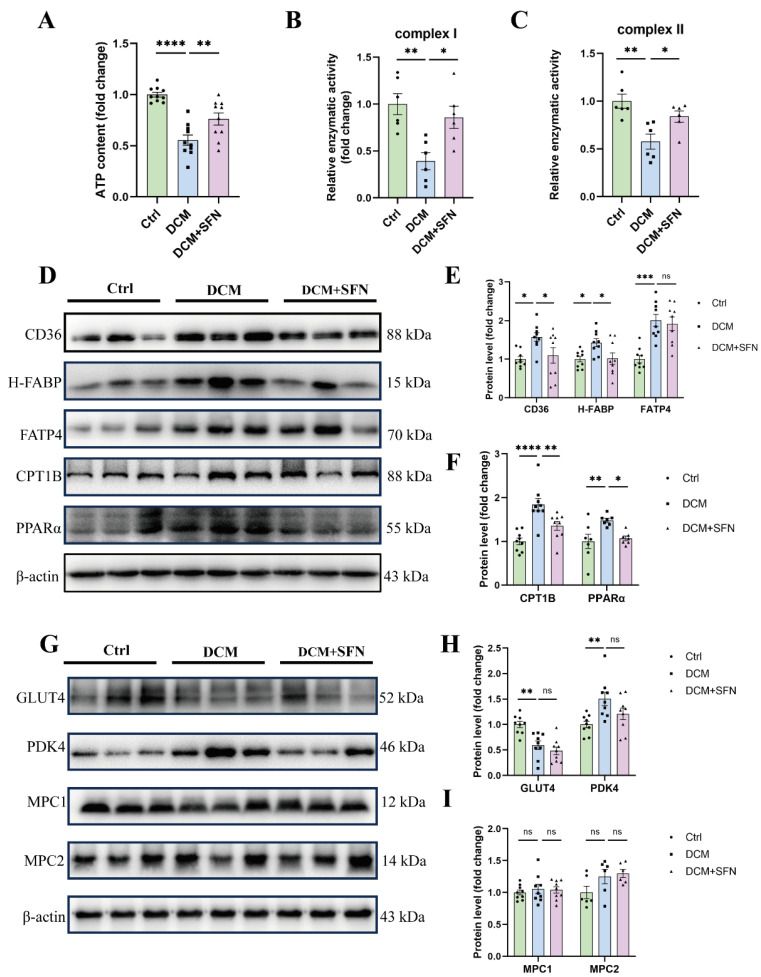
SFN-I improves cardiac mitochondrial function and modulates key regulators of FA metabolism, rather than glucose metabolism in db/db mice. (**A**–**C**) Cardiac ATP content and the activities of mitochondrial complexes I and II. (**D**–**I**) Representative Western blot images and relative quantification of proteins related to cardiac metabolism in heart tissues. Data are expressed as the mean ± SEM (n = 6–9). * *p* < 0.05, ** *p* < 0.01, *** *p* < 0.001, **** *p* < 0.0001: significantly different as indicated; ns: not significantly different. CD36, cluster of differentiation 36; H-FABP, heart-type fatty acid-binding protein; FATP4, fatty acid transport protein 4; CPT1B, carnitine palmitoyltransferase 1B; PPARα, peroxisome proliferator-activated receptor alpha; GLUT4, glucose transporter type 4; PDK4, pyruvate dehydrogenase kinase 4; MPC1/2, mitochondrial pyruvate carrier 1/2; Ctrl, control; DCM, diabetic cardiomyopathy; SFN, sulforaphane.

Our results showed a significant decrease in cardiac ATP production and the activity of mitochondrial complexes I and II in the db/db model mice compared with that in the control mice. Importantly, those abnormalities were significantly attenuated by SFN-I (Figure 6A–C). To further explore how SFN-I improves cardiac energy metabolism, we measured the expression of key proteins that are associated with cardiac glycolipid metabolism, including transcellular transporters for FAs (CD36, H-FABP, and FATP4) and glucose (GLUT4), the transmitochondrial transporter for FAs and the rate-limiting enzyme for FA oxidation (CPT1B), the mitochondrial pyruvate carriers (MPC1 and MPC2), the pyruvate oxidation inhibitor (PDK4), and the master sensor and regulator of metabolism (PPARα). Western blot analysis indicated that the protein expression levels of CD36, H-FABP, FATP4, CPT1B, and PPARα were significantly increased in db/db mice, all of which, except for FATP4, were significantly inhibited by SFN-I (Figure 6D–F). Regarding glucose metabolism, decreased GLUT4, increased PDK4, and no significant alterations in MPC1 and MPC2 were observed in the heart of db/db model mice compared with control mice. SFN-I induced a reduction in PDK4 expression, but this difference was not significant. The levels of GLUT4, MPC1, and MPC2 also showed no significant differences following SFN-I (Figure 6G–I).

## 4. Discussion

In this study, a db/db male mouse T2DM-DCM model was utilized for testing the effect of SFN-I, in the form of oral gavage with 10 mg/kg every other day for a 16 week period. With the current experimental settings, the model group exhibited characteristic T2DM cardiac remodeling marked by reduced diastolic function but preserved systolic function, cardiac injury, and fibrosis, without significant hypertrophy or dilation. SFN-I mitigated this remodeling through mechanisms involving improvements in systemic and cardiac metabolism. Systemically, the intervention effectively alleviated hyperglycemia, HOMA-IR, hyperlipidemia, and serum MDA levels, reduced NAS and liver TNF-α levels, while having no appreciable effect on adiposity, serum insulin levels, or liver IL-6, TG, and TC levels. Locally, the intervention inhibited cardiac lipid deposition and peroxidation, as well as superoxide anion formation, and prevented the reduction in mitochondrial complex I and II activities and ATP production, but did not influence the elevated ceramide levels. Correspondingly, the db/db mice showed a marked shift in cardiac substrate utilization towards FAs, as indicated by altered protein expressions of multiple key metabolic mediators; SFN-I significantly modulated those associated with FA metabolism but not with glucose metabolism.

Systemic metabolic disorders are significant risk factors for DCM. They determine substrate accessibility, contribute to local IR, and activate a profibrotic response by affecting various cell types and extracellular matrix (ECM) in the heart [2]. In this study, SFN-I notably improved hyperglycemia, HOMA-IR, hyperlipidemia, and serum MDA in db/db mice, but not BW or body lean and fat mass. Yet serum IL-6 and TNF-α levels did not change significantly among the three groups (Figure 1 and Table 1). The hypoglycemic, hypolipidemic, and insulin-sensitizing activities of ITCs, particularly SFN, have been demonstrated in both CMD animal models and patients, though the findings were inconsistent [21,22,23,24,27,28,29,30,31,32,33,34,36,37,38,39,40,41,42,44,45,46,47,49,50,55,56,57]. Obesity and T2DM are intricately interconnected. The anti-obesity or weight-loss properties of ITCs and ITC-rich extracts have been discovered in some preclinical studies. Nevertheless, the findings remain controversial across distinct models, with inconsistent data being reported in db/db mice [32,33,34,38,39,40,41,42,43,44,45,46,47,48,50,53,55,56,57,58]. The anti-obesity effect of SFN involves modulating liver and adipose tissue metabolism. Notably, this efficacy is accompanied by a reduction in leptin levels and may even rely on functional leptin receptor signaling [32,46,59], offering a potential explanation for our negative results observed in db/db mice. The liver is the central metabolic organ, and MAFLD is an independent risk factor for CMD. We found that SFN-I significantly ameliorated liver histopathology, as determined by the reduced total NAS, lobular inflammation score, and TNF-α expression (Figure 2B,D,G). Although SFN-I induced a decreasing trend in steatosis score and levels of TG, TC, and IL-6, the difference was not statistically significant (Figure 2C,2F,I,J). The elevated ballooning score was not affected by SFN-I (Figure 2E). The protective effect of SFN on MAFLD has been reported in HFD-fed mice [32,33,34,35,36,37,38,39,40,41,42]. Moreover, easing MAFLD may contribute to the antidiabetic function of SFN [60]. To sum up, our findings demonstrate that SFN-I can improve the overall metabolic state of db/db mice, notwithstanding the lack of remission in BW, body composition, and liver lipid accumulation.

Cardiac remodeling is the main characteristic and subclinical phenotype of various CMDs and also a proposed surrogate endpoint for major adverse cardiovascular events. Hence, a comprehensive assessment of it was conducted herein. At the end of the experiment, the 25-week-old db/db mice exhibited preserved systolic function but reduced diastolic function. Simultaneously, cardiac fibrosis, architectural abnormalities, and inflammatory cell infiltration were evident. The HW/TL ratio and cardiomyocyte areas increased; however, only the former reached statistical significance. Unexpectedly, no significant differences in LV wall thickness or diameters were observed. These findings imply that the hearts of the model mice exhibited a characteristic fibrotic phenotype without significant hypertrophy or dilation. Notably, both this phenotype and the concomitant diastolic dysfunction showed marked improvement following SFN-I (Figure 3 and Figure 4, and Table 2). The extent of cardiac fibrosis has been shown to positively correlate with mortality and HF-related hospitalization rate, and predict poor prognosis in diabetic patients [1,2]. Diastolic and systolic dysfunctions are actually more typical in T2DM and T1DM, respectively, though considered sequential in DCM progression [1,2]. Two main HF phenotypes have been identified: restrictive/HF with preserved EF (HFpEF) and dilated/HF with reduced EF (HFrEF). Most clinical reports concerning DCM conform to the former phenotype, which is in line with the fact that many HF patients have normal LV EF and size [2,3]. Our fibrosis results fit the trait in HFpEF (moderate diffuse fibrosis) but not that observed in HFrEF (larger areas of fibrosis secondary to cardiomyocyte injury and death) [3]. The protection of SFN on DCM has been documented in various diabetes models, though their disease phenotypes and degree of improvement are not completely consistent with ours [48,49,50,51,52,53,54]. The differences might be ascribed to variations in animal models, disease progression, SFN dosage, and intervention modalities. Our results indicate that the db/db mouse model is a relatively reliable system for studying DCM, as it develops a typical cardiac remodeling phenotype similar to T2DM patients. Using this model, we provide solid evidence that chronic oral SFN-I significantly alleviates DM-induced cardiac diastolic dysfunction and histopathological alterations.

The alteration in cardiac metabolism, whether at physiological or pathological settings, is causative in cardiac remodeling [10]. Cardiac lipotoxicity represents a hallmark and mechanistic underpinning of T2DM cardiomyopathy. It results from excessive lipid accumulation and subsequent production of lipotoxic metabolites, particularly ceramide and DAG, which may mediate IR, inflammation, membrane destabilization, and apoptosis [1,2,3,4,6,7,8,9]. Intramyocardial lipid deposition is suggested to be an independent predictor for diastolic dysfunction in patients with T2DM and is associated with adverse cardiovascular events [2,8]. Herein, SFN-I effectively reduced it in db/db mice (Figure 5A,B), consistent with previous observations in HFD + STZ-treated mice [49,53]. Besides, it was ceramide, rather than DAG, that showed a significant increase in the model group; however, SFN-I had no significant attenuation effect (Figure 5C,D). To date, no studies have explored the impact of ITCs on cardiotoxic lipid metabolites. However, Teng et al. reported that SFN mitigated hepatic ceramide accumulation both in vitro and in vivo, probably by inhibiting the de novo synthesis pathway [61,62].

Myocardial oxidative stress and mitochondrial dysfunction are associated with lipotoxicity, particularly in DCM, and are even regarded as its primary mediators [1,2]. We observed increased production of superoxide anions and MDA, reduced ATP content, and impaired activities of mitochondrial complex I and II in the hearts of db/db mice, which were all significantly reversed by SFN-I (Figure 5E–G and Figure 6A–C). SFN was shown to activate Nrf2 signaling to mitigate oxidative stress in various diseases. Nrf2 and its downstream MT also mediated the preventive effect of SFN on DCM, and may underlie the synergistic effect of SFN and zinc in DCM [48,49,50,51,52,53,54,63]. The modulation of mitochondrial functionality and integrity by SFN has also been documented [64,65,66,67,68,69]. However, relevant studies specifically addressing DCM are limited, with only indirect evidence suggesting the induction of peroxisome proliferator-activated receptor gamma coactivator-1 alpha (PGC-1α) [53]. Mitochondrial energetics is integral to its pleiotropic functions, and its abnormalities may arise from a series of interlinked events. These include metabolic disorders, Ca2^+^ mishandling, oxidative stress, altered expression and modification of electron transport chain (ETC) components, mitochondrial dynamics imbalance, and impairments in biogenesis and mitophagy [12,13]. All these factors are reportedly modulated by SFN, deserving further investigation in DCM. Taken together, our data from db/db mice indicate that SFN-I significantly ameliorates cardiac lipotoxicity, as evidenced by reduced lipid accumulation and oxidative stress, and improved mitochondrial function, despite no observed favorable effects on ceramide generation.

Lipotoxicity-causing altered cardiac metabolism under DCM is marked by increased but unmatched FA uptake and oxidation. This seems to be ascribed to higher substrate embedding (mainly derived from circulating free FAs and TG) and impaired substrate metabolism adjustment [1,2,7,8,10]. Improving blood lipid profiles and systemic IR by SFN has been reported previously [28,29,30,31], and was also observed herein. Does this mean that the protection of SFN against DCM involves modulating cardiac substrate metabolism? Transport of FAs is crucial for cardiac FA utilization and is primarily mediated by specific transporters associated with PPARα [18,19]. We show significant up-regulation of CD36, H-FABP, FATP4, CPT1B, and PPARα in the myocardial tissues of the model group, all of which, except for FATP4, were reversed or alleviated by SFN-I (Figure 6D–F). Increased CD36 expression has been found on the cardiac sarcolemma of some lipotoxic cardiomyopathy models and in cardiomyocytes of DCM patients [5]. It accounts for most cardiac FA uptake, either in conjunction with FABP or FATP or independently [6], and also disturbs metabolism by inducing a detrimental mitochondrial metabolism switch and impairing AMPK activity [5]. Despite limited research references, our team and others have shown that SFN-I induces downregulated CD36 expression and ectopic lipid accumulation in the arterial walls of apoE^−/−^ mice and in the kidneys of unilateral ureteral obstruction mice, but does not affect CD36 expression in the hearts of HFD + STZ-treated mice [53,70,71]. The effects and implications of ITCs on FABP and FATP are largely unknown, except for one study showing that SFN inhibits FABP4 expression in hormone-treated mouse embryonic fibroblasts [72]. Our data on PPARα, CPT1B, CD36, and FATP4 in db/db mice vs. control mice are in line with previous findings [73]. Interestingly, in HFD + STZ-treated mice, AMPK-mediated lipid metabolism proved pivotal for defense against DCM by SFN, where reversed DM-caused declines in PPARα and CPT1B were observed, a finding opposite to ours [53].

Inhibiting cardiomyocyte FA metabolism, especially its uptake, is thought to be beneficial for DCM. However, it is ambiguous whether to restrict or promote oxidation. Altered cardiac FAO is adaptive in the short term but maladaptive and harmful to cardiac function in the long term. Yet, reduced and impaired FAO may also affect ATP production and cause lipotoxic intermediate accumulation [2,7,8,15]. Therefore, the outcome of manipulating FA metabolism is likely pathway-specific and requires considering FA overload, disease types, and stages [6,7,8]. Our results suggest that improving myocardial lipotoxicity by SFN-I might involve inhibiting FA metabolism mediated by PPARα-related CD36, H-FABP, and CPT1B expression.

Impaired glucose utilization and IR are also implicated in the pathogenesis of DCM. FAs and glucose may compete for cardiac catabolism [1,4,6]. SFN can antagonize IR and improve glucose metabolism in the liver, adipose tissue, and muscle [43,74,75,76,77,78,79]. In turn, glucose availability proves to be a decisive factor for activating Nrf2 [80]. Both SFN and BSE prevent cardiac glycogen accumulation in db/db mice [50]. One may ask whether SFN-induced inhibition on FA metabolism coincides with increased glucose metabolism? Hence, we conducted our preliminary assessment. As previously reported, a reduced abundance of GLUT4 was observed in db/db mice compared with that in control mice [73]. Unexpectedly, SFN-I generated no difference (Figure 6G,H). Indeed, several studies have explored the impact of ITCs on GLUT4 in non-cardiac tissues in CMD models. Most of them have found a stimulatory role related to insulin sensitivity and adipocyte browning, with one exception [74,75,76,77,78,79].

Both MPC and PDK function as hubs in glucose aerobic oxidation. MPC gates the entry of pyruvate into the mitochondria as its sole carrier, while PDK inactivates PDH through phosphorylation [15,16,17]. Our results show elevated PDK4 levels in db/db mice compared to WT mice. SFN-I reduced this elevation, but not significantly. Additionally, MPC1 and MPC2 levels showed no statistically significant differences among groups (Figure 6G,I). Elevated PDK levels and activities have been identified in the tissues of patients and mammals afflicted with CMD, correlating with poor glucose disposal, excessive FA utilization, and cardiac dysfunction [10,81,82]. Conversely, a reduction in MPC abundance and activity has been observed in the hearts of HF patients and Akita diabetic mice, likely contributing to adverse cardiac remodeling and dysfunction [83,84,85]. As a relatively new metabolic regulator, MPC has not been investigated in the context of ITCs, and there is limited knowledge regarding classical PDK as well. Based on these player expressions, our present observations imply that impaired myocardial glucose metabolism might coexist with lipotoxicity in db/db mice due to dysregulated Glut4 and PDK4 rather than MPC1 and MPC2; however, SFN-I appears to have little influence on this process. These issues deserve further study.

### Limitations

First, the study utilized only male mice and a single dose of SFN. Future research should incorporate both sexes for comparative analysis and should evaluate an effective dose range along with dose–response relationships and the threshold for toxic or side effects. Second, this is an observational study primarily focused on the endpoint. To draw more rigorous causal conclusions, time-series evaluations of key indices and genetic manipulations are necessary. Third, when exploring cardiac metabolism, we indirectly assess it by analyzing the total expression levels of multiple key regulators via Western blotting. Direct assessment using more refined approaches like substrate labeling and metabolomics, as well as more precise quantification of the expression and modification of these key regulators, their subcellular distribution, translocation, and activities, and other important metabolism-related effectors and mediators, warrants further investigation. Fourth, constrained by the limited availability of heart tissues, some indicators lack validation across different methodologies; some indicators or sub-indicators lack focus, such as inflammatory reactions (quantitation and specificity of inflammatory cells and mediators, etc.), microvascular abnormalities (rarefaction, EC dysfunction, perivascular fibrosis, etc.), sources of ROS, mitochondrial morphology, turnover, and surveillance. Lastly, although myocardiocytes constitute the majority, the heart exhibits cellular heterogeneity, and these cells may interact and collectively contribute to DCM. Therefore, myocardiocyte-specific analysis combined with multicellular targets and intercellular communication is imperative.

## 5. Conclusions

**Figure 7:**
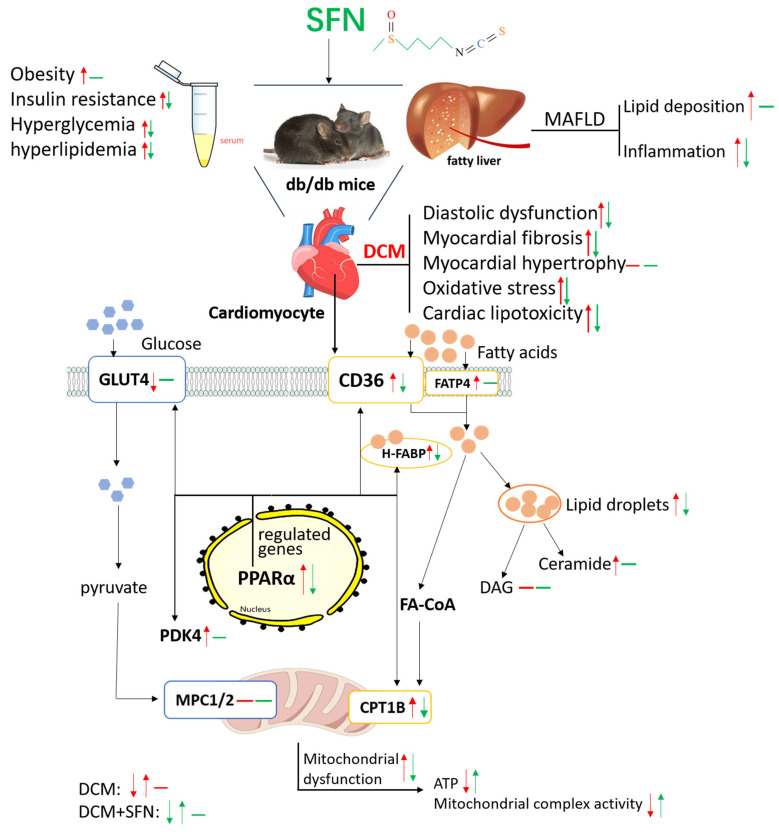
Schematics depicting the effects and underlying mechanisms of SFN-I on DCM. SFN, sulforaphane; DCM, diabetic cardiomyopathy; MAFLD, metabolic dysfunction-associated fatty liver disease; NAS, NAFLD activity score; FAs, fatty acids; GLUT4, glucose transporter type 4; CD36, cluster of differentiation 36; FATP4, fatty acid transport protein 4; H-FABP, heart-type fatty acid binding protein; FA-CoA, fatty acid-coenzyme A; CPT1B, carnitine palmitoyltransferase 1B; PPARα, peroxisome proliferator-activated receptor alpha; PDK4, pyruvate dehydrogenase kinase 4; MPC1/2, mitochondrial pyruvate carrier 1/2; Ctrl, control; DAG, diacylglycerol; ↑↑, increase; ↓↓, decrease; ——, no change; ↓↑—, DCM; ↓↑—, DCM + SFN.

In conclusion, our current study suggests that chronic oral administration of SFN in male T2DM db/db mice protects against DCM by mitigating overall metabolic dysregulation and inhibiting cardiolipotoxicity. The latter might involve controlling cardiac FA metabolism and improving mitochondrial function, rather than promoting glucose metabolism (Figure 7). Our findings provide robust preclinical evidence for the protective effect of SFN-I against DCM and offer comprehensive and novel mechanistic insights into this effectiveness. These results, together with the existing literature [48,49,50,51,52,53,54], underscore the need for further studies with stronger evidence, including clinical trials and cohort studies, as well as specific and precise assessments of cardiac metabolic flexibility and mitochondrial function. Such investigations would not only further highlight the health advantages of consuming cruciferous vegetables but also support the potential application of SFN supplementation or related agents in DCM management.

## Figures and Tables

**Table 1:** SFN-I improves serum lipid profile and MDA levels in db/db mice.

Groups	Ctrl	DCM	DCM + SFN
FFAs (mmol/L)	0.44 ± 0.03	1.11 ± 0.08 ****	0.80 ± 0.08 **
TC (mmol/L)	2.25 ± 0.21	4.85 ± 0.63 **	2.97 ± 0.47 *
TG (mmol/L)	0.52 ± 0.04	1.30 ± 0.10 ****	1.10 ± 0.09
LDL-C (mmol/L)	0.49 ± 0.05	1.45 ± 0.25 **	1.07 ± 0.26
HDL-C (mmol/L)	1.06 ± 0.26	0.96 ± 0.60	1.66 ± 0.54 *
MDA (μM)	6.42 ± 0.37	13.31 ± 0.62 ****	9.46 ± 0.64 ***
IL-6 (pg/mL)	112.6 ± 6.28	109.0 ± 5.64	112.7 ± 4.23
TNF-α (pg/mL)	743.9 ±25.40	748.5 ± 40.90	793.6 ± 9.71

Data are expressed as the mean ± SEM (n = 6–10). Abbreviations: FFAs, free fatty acids; TC, total cholesterol; TG, total triglycerides; LDL-C, low-density lipoprotein cholesterol; HDL-C, high-density lipoprotein cholesterol; MDA, malondialdehyde; IL-6, interleukin-6; TNF-α, tumor necrosis factor-alpha; Ctrl, control; DCM, diabetic cardiomyopathy; SFN, sulforaphane. ** *p* < 0.01, **** *p* < 0.0001 vs. Ctrl group. * *p* < 0.05, ** *p* < 0.01, *** *p* < 0.001 vs. DCM group.

**Table 2:** Left ventricular diameter and wall thickness in three groups at week 16.

Groups	Ctrl	DCM	DCM + SFN
LVIDs (mm)	2.23 ± 0.10	1.91 ± 0.14	2.04 ± 0.11
LVIDd (mm)	3.67 ± 0.09	3.53 ± 0.11	3.80 ± 0.12
LVAWs (mm)	1.61 ± 0.08	1.75 ± 0.10	1.71 ± 0.06
LVAWd (mm)	1.08 ± 0.04	1.10 ± 0.05	1.02 ± 0.05
LVPWs (mm)	1.54 ± 0.07	1.66 ± 0.12	1.55 ± 0.07
LVPWd (mm)	1.00 ± 0.06	1.11 ± 0.09	1.01 ± 0.06

Data are expressed as the mean ± SEM (n = 8–10). Abbreviations: LVIDs/d, left ventricular internal diameter at end-systole and end-diastole; LVAWs/d, left ventricular anterior wall thickness at end-systole and end-diastole; LVPWs/d, left ventricular posterior wall thickness at end-systole and end-diastole; Ctrl, control; DCM, diabetic cardiomyopathy; SFN, sulforaphane. No statistical significance was found among groups by one-way ANOVA analysis with Bonferroni’s multiple comparisons test.

**Table 3:** Blood pressure in three groups at week 16.

Groups	Ctrl	DCM	DCM + SFN
HR (bpm)	670 ± 29	562 ± 43	606 ± 15
SBP (mmHg)	125 ± 4	122 ± 8	124 ± 4
DBP (mmHg)	79 ± 3	72 ± 9	85 ± 7
MBP (mmHg)	95 ± 3	89 ± 9	98 ± 6

Data are expressed as the mean ± SEM (n = 4–7). Abbreviations: HR, heart rate; SBP, systolic blood pressure; DBP, diastolic blood pressure; MBP, mean blood pressure; Ctrl, control; DCM, diabetic cardiomyopathy; SFN, sulforaphane. No statistical significance was found among groups by one-way ANOVA analysis with Bonferroni’s multiple comparisons test.

## Data Availability

The data are contained within the article.

## Abbreviations

The following abbreviations are used in this manuscript:

SFN	Sulforaphane
SFN-I	Sulforaphane intervention
ITC	Isothiocyanate
DCM	Diabetic cardiomyopathy
T2DM	Type 2 diabetes mellitus
WT	Wild-type
MAFLD	Metabolic dysfunction-associated fatty liver disease
NAFLD	Nonalcoholic fatty liver disease
CMD	Cardiometabolic diseases
NAS	Nonalcoholic fatty liver disease activity score
DM	Diabetes mellitus
HF	Heart failure
IR	Insulin resistance
HOMA-IR	Homeostasis model assessment of insulin resistance
ER	Endoplasmic reticulum
MC	Metabolic cardiomyopathy
FA	Fatty acid
CD36	Cluster of differentiation 36
H-FABP	Heart-type fatty acid binding protein
FATP4	Fatty acid transport protein 4
CPT1B	Carnitine palmitoyltransferase 1B
PPARα	Peroxisome proliferator-activated receptor alpha
PDK4	Pyruvate dehydrogenase kinase 4
GLUT4	Glucose transporter type 4
MPC1	Mitochondrial pyruvate carrier 1
MPC2	Mitochondrial pyruvate carrier 2
PDH	Pyruvate dehydrogenase
FAO	Fatty acid beta oxidation
FGF21	Fibroblast growth factor 21
WAT	White adipose tissue
Nrf2	Nuclear factor E2-related factor 2
AMPK	AMP-activated protein kinase
BSE	Broccoli Sprout Extract
DAG	Diacylglycerol
ROS	Reactive oxygen species
ATP	Adenosine triphosphate
EC	Extracellular matrix
CVDs	Cardiovascular diseases
LV	Left ventricle
HFpEF	HF with preserved EF
HFrEF	HF with reduced EF
PGC-1α	Peroxisome proliferator-activated receptor gamma coactivator-1 alpha
ETC	Electron transport chain
HFD	High-fat diet
FFAs	Free fatty acids
TC	Total cholesterol
TG	Triglyceride
HDL-C	High-density lipoprotein-cholesterol
LDL-C	Low-density lipoprotein-cholesterol
IL-6	Interleukin-6
TNF-α	Tumor necrosis factor Alpha
MDA	Malondialdehyde
INS	Insulin
WGA	Wheat germ agglutinin
H&E	Hematoxylin and eosin
DHE	Dihydroethidium
HW/TL	Heart weight to tibia length
BW	Body weight
OCT	Optimum cutting temperature
ELISA	Enzyme-linked immunosorbent assay
HR	Heart rate
SBP	Systolic blood pressure
DBP	Diastolic blood pressure
MBP	Mean blood pressure
LVIDs/d	Left ventricular internal diameter at end-systole and end-diastole
LVAWs/d	Left ventricular anterior wall thickness at end-systole and end-diastole
LVPWs/d	Left ventricular posterior wall thickness at end-systole and end-diastole
EF	Ejection fraction
FS	Fractional shortening
E/A	Early-to-atrial filling ratio
AET	Aortic ejection time
IVCT	Isovolumic contraction time
IVRT	Isovolumic relaxation time
MPI	Myocardial performance index
